# Inducing human parthenogenetic embryonic stem cells into islet-like clusters

**DOI:** 10.3892/mmr.2014.2588

**Published:** 2014-09-22

**Authors:** JIN LI, JINGJING HE, GE LIN, GUANGXIU LU

**Affiliations:** 1 Institute of Reproductive and Stem Cell Engineering, Central South University, Changsha, Hunan 410078, P.R. China; 2 National Engineering Research Center of Human Stem Cells, Changsha, Hunan 410078, P.R. China

**Keywords:** Human parthenogenetic embryonic stem cells, induction, islet, imprinted gene

## Abstract

In order to determine whether human parthenogenetic embryonic stem (hpES) cells have the potential to differentiate into functional cells, a modified four-step protocol was used to induce the hpES cells into islet-like clusters (ILCs) *in vitro*. Growth factors activin A, retinoic acid, nicotinamide, Exendin-4 and betacellulin were added sequentially to the hpES cells at each step. The terminally differentiated cells were shown to gather into ILCs. Immunohistochemistry and semi quantitative polymerase chain reaction analyses demonstrated that the ILCs expressed islet specific hormones and functional markers. Furthermore, an insulin release test indicated that the clusters had the same physiological function as islets. The ILCs derived from hpES cells shared similar characteristics with islets. These results indicate that hpES cell-derived ILCs may be used as reliable material for the treatment of type I diabetes mellitus.

## Introduction

The process of parthenogenesis is the development an egg into an embryo without the participation of sperm. This form of reproduction exists in reptiles, fish and birds, but not in mammals. A mammalian parthenogenetic embryo cannot develop into a fetus and is usually arrested at a particular developmental stage (1). This developmental arrest probably correlates to a lack of paternally imprinted genes (1,2). However, the eggs of mammals can be activated *in vitro* and develop into blastocysts. The inner cell mass may be used to create embryonic stem cells (ESCs), known as parthenogenetic embryonic stem cells (pESCs). Previous studies (3,4) have reported that pESCs have been successfully established in rats and primates. Revazova *et al* (5) reported the first successful derivation of human pESCs (hpESCs) in 2007, following this, numerous groups have also achieved hpESC derivation (6, 7).

A major concern ahead of the clinical utilization of hpESCs is whether these cells hold the same differentiation ability as normal hESCs; as a chimerical study showed that pESCs only exist in limited tissues (8). Therefore the differentiation potential of hpESCs into specific terminal cells needs to be tested further. Numerous data have shown that pESCs from rodents and non-human primates can differentiate into mid-brain dihydroxyphenyl-ethylamine neurons (9), hepatic endoderms, hematopoietic cells including CD45+ cells, lymphocytes, monocytes and akaryocyte-like cells (10), cardiocytes, lipocytes and epithelium (11).

pESCs are derived entirely from maternal genes. It was previously determined that there is a correlation between the expression of imprinted genes and the likelihood whether pESCs will develop into functional tissues or not (12). Due to the lack of paternal genes, pESCs do not express paternally imprinted genes, including insulin-like growth factor 2 (IGF2), which is the main growth factor involved in the promotion of mitosis (13). The expression of IGF2 is also essential for the long-term proliferation of all cell types (14). Previously, the endogenous expression levels of IGF2 were shown to be varied between mouse pESCs, androgenetic ESCs and normal ESCs (11). In addition, studies have shown that the differentiation potential of pESCs from nuclear transplantation *in vivo* and *in vitro* were significantly enhanced as compared with primitive pESCs (~2–5 times) (15). Therefore, it needs to be confirmed whether the differentiation potential of hpESCs differs from that of hESCs.

Therefore, the present study aimed to confirm whether hpESCs can be induced into islet-like clusters (ILCs) and compare the difference between normal ESCs and hpESCs in this differentiation progress.

## Materials and methods

### Culture and differentiation

**Figure 1:**
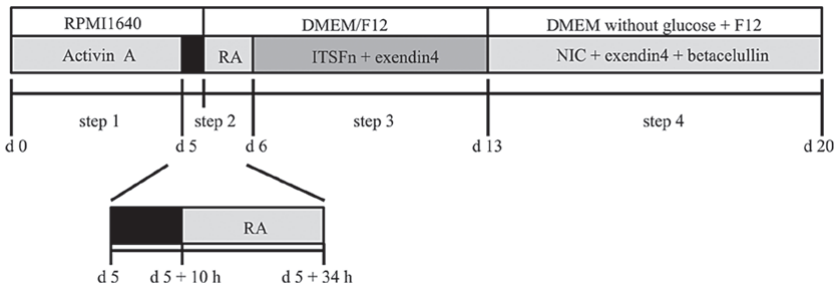
Schematic diagram of the four-step method used to induce human parthenogenetic embryonic stem cells into islet-like clusters. The differentiation protocol was divided into four steps and the growth factors, medium and length of duration for each step are shown. The black box represents the removal of Activin A and serum followed by culturing the cells in RPMI-1640 medium alone for 10 hours. RPMI 1640, Roswell Park Memorial Institute 1640 medium; DMEM/F12, Dulbecco’s Modified Eagle’s Medium/Nutrient Mixture F12; NIC, nicotinamide; RA, retinoic acid; d, day; h, hour.

The present study was approved by the Ethics Committee of Central South University, Changsha, China. Undifferentiated chHES8 (normal ESCs), chHES32 (hpESCs) and chHES69 (hpESCs) were established, according to previous methods (16), and were maintained on human embryonic fibroblasts in Dulbecco’s Modified Eagle Medium/Nutrient Mixture F12 (DMEM/F12) (Invitrogen Life Technologies, Carlsbad, CA, USA) supplemented with 15% (vol/vol) KnockOut™ serum replacement, 1 mM non-essential amino acids, Glutamax™, 0.1 mM β-mercaptoethanol (Invitrogen Life Technologies), and 4 ng/ml recombinant human fibroblast growth factor (FGF2) (Invitrogen Life Technologies, Minneapolis, MN, USA). Cultures were manually passaged at a 1:4 ratio at seven day intervals, and began to differentiate on the fifth day following the last passage. The induction protocol was divided into four steps. (i) Activin A (100 ng/ml, R&D Systems, Minneapolis, MN, USA) and low dosage Hyclone™ serum (GE Healthcare, South Logan, UT, USA) were used to generate definitive endoderm (DE) from hpESCs for five days in the first stage of the process (17). (ii) Following withdrawal of the Activin A and serum, the cells were cultured in RPMI-1640 medium for an interval of 10 hours, followed by the addition of retinoic acid (RA, 10^−5^ M, Sigma-Aldrich, St Louis, MO, USA) for 24 hours to initiate the pancreatic lineage specification. (iii) A mixture of 1% ITS (100x), fibronectin (5 μg/ml) and Exendin-4 (Ex-4, 50 ng/ml, Sigma-Aldrich) were added to the medium for one week, in order to further differentiate the cells into pancreatic precursor cells. (iv) The pancreatic precursor cells were cultured in suspension with a medium containing 1% N_2_ (100×), 1% B27 (50x, Gibco-BRL, Carlsbad, CA, USA), nicotinamide (NIC, 10^−2^ M), Ex-4 (50 ng/ml) and betacellulin (20ng/ml, R&D Systems) for an additional week to obtain insulin producing cells. The induction method can be seen in Fig. 1.

### Semi quantitative polymerase chain reaction (qPCR)

Total RNA was extracted using the TRIzol^®^ reagent (Invitrogen Life Technologies) and cDNA was synthesized from 1 μg of total RNA using random primers and the Reverse Transcriptase kit (Thermo Fisher Scientific, Rockford, IL, USA). All PCR reactions were performed using Taq DNA polymerase with various annealing temperatures and cycle numbers in a total reaction volume of 10 μL. PCR products were separated using 2% agarose gels and visualized with ethidium bromide staining. The primer pairs and expected amplicon sizes are listed in Table 1.

### Immunofluorescence staining

The cells were harvested on days 5, 13, and 20, and were fixed in phosphate-buffered saline (PBS) containing 4% paraformaldehyde for 15 min at room temperature, followed by three washes with PBS containing 0.1% bovine serum albumin (BSA). The cells were permeabilized using 0.1% Triton X-100 in PBS containing 0.1% BSA and 4% normal goat serum (Gibco-BRL), or 10% donkey serum for Sox17. The cells were incubated with the primary antibodies overnight at 4ºC, followed by a 1 h incubation with the secondary antibodies at room temperature. The following antibodies and dilutions were used: Goat anti-human sox17, 1:40 (R&D Systems); guinea pig anti-human pdx1, 1:200 (Abcam, Cambridge, MA, USA); mouse anti-human insulin l:200 (Sigma-Aldrich). Donkey anti-goat antibody was used at 1:300 (Sigma-Aldrich); goat anti-guinea pig antibody was used at 1:500 (abcam) and fluorescein isothiocyanate anti-human insulin antibody was used at 1:400 (Chemicon, Temecula, CA, USA). The cells were mounted in ten random fields of vision using DAPI (BD Biosciences, Franklin Lakes, NJ, USA) dye, and examined using a fluorescence microscope (Nikon Inc., Melville, NY, USA). Each vision contained >200 cells and totaled >2000 cells per sample.

### Insulin release assay

The ILCs were transferred into a four-well dish and the number of clusters was recorded. The clusters were washed twice with Hank’s Balanced Salt Solution (HBSS) containing 0.5% human serum albumin (HSA), for 10 minutes each time. Following the wash steps, the clusters were pre-incubated for 30 minutes in medium containing 5.5 mM glucose. Subsequently, the clusters were incubated in 5.5 and 25.0 mM glucose for one hour and stored at −20ºC. The insulin released into the medium was detected using the Insulin kit (12017547; Roche Diagnostics GmbH, Mannheim, Germany) and Roche E170 equipment. According to the standard curve, the detection range was between 2.6 and 24.9μU/ml.

### Proliferation assay

The cells were harvested on differentiation days 5, 13 and 20, and fixed in PBS containing 4% paraformaldehyde for 15 min, followed by three washes with PBS containing 0.1% BSA. The cells were incubated with anti-Ki67 antibody (mouse anti-human, Sigma-Aldrich, dilution 1:10) for 30 min at room temperature. Following the initial incubation with the primary antibody, the cells were incubated with the appropriate secondary antibody, as described previously. The cells were mounted in 10 random fields of vision using DAPI. Each vision contained >200 cells and totalled >2000 cells/sample.

### Statistical analyses

All experiments were repeated three times and data are expressed as the mean±standard deviation. Statistical analyses were performed using the Student’s t-test. P<0.05 was considered to indicate a statistically significant difference.

## Results

### Generation of definitive endoderm (DE) cells

**Figure 2:**
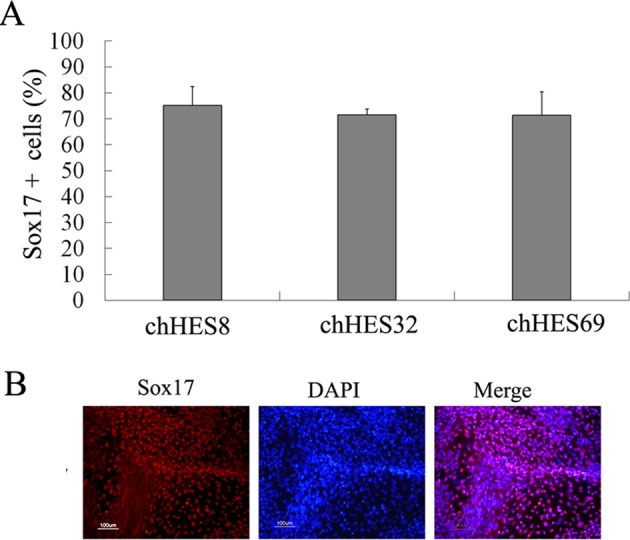
Generation of DE cells. (A) Expression levels of Sox17 on day 5 in the three embryonic stem cell lines.(B) DE marker sox17 was detected on day 5 by immunofluorescence analysis. The nuclei were stained with DAPI. Data are expressed as the mean ±standard deviation. Scale bar: 100 μm. chHES8, normal human ES; chHES32 and chHES69, human parthenogenetic ES; ES, embryonic stem cells; DE, definitive endoderm.

HpESCs were treated with 100 ng/ml Activin A and a low concentration of serum in order to induce DE. After 5 days, endoderm-specific Sox17 and Foxa2 genes were shown to be expressed. Expression of the mesoderm-related gene brachyury, and the ectoderm-related gene Pax6 were not detected on day 5. Furthermore, there were no pancreatic-related genes detected. The DE marker Sox17 reached ~71.6±2.1% on day 5 (Fig. 2A and B)

### Differentiation of pancreatic precursor cells

**Figure 3:**
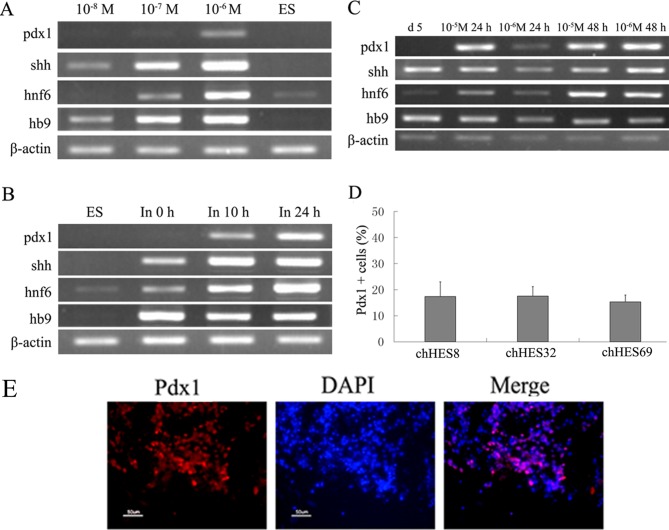
Differentiation of PP cells. (A–C) Effects of RA in pancreatic specialization. (A) Gene expression in response to RA treatment at different concentrations for 24 h following Activin A treatment for 5 days and 10 h treatment with L-glutamine. (B) Gene expression in response to 24 h 10 6 M RA treatment at different time intervals. (C) Gene expression in response to 24 and 48 h treatments with 10^−5^ M and 10^−6^ M RA for 10 h. (D) Expression levels of Pdx1 on day 13 in the three embryonic stem cell lines. (E) PP marker Pdx1 was detected on day 13 by immunofluorescence analysis. The nuclei were stained with DAPI. Data are expressed as the mean ± standard deviation. Scale bar: 50um. ES, embryonic stem cells; PP, pancreatic precursor; RA, retinoic acid.

After treatment with Activin A for 5 days, cells were transferred into media containing L-glutamine and RPMI-1640 for ~10 hours. Fresh media containing DMEM/F12 and RA was used to initiate of pancreatic specialization. Retinoic acid (RA) was used at several concentrations (10^−4^, 10^−5^, 10^−6^, 10^−7^ and 10^−8^ M). RA was shown to rapidly upregulate the gene expression levels of Pdx1 when the concentration of the RA was >10^−6^ M. However, at concentrations approaching 10^−4^ M, cell death was observed and the cultures could not continue (data not shown). Suitable concentrations of RA were chosen (10^−5^ M and 10^−6^ M) to determine the gene expression levels of Pdx1 over varying durations (24 and 48 hours). Following a 24 hour period of treatment, the gene expression levels of Pdx1, in the cells treated with 10^−5^ M RA was stronger as compared with the expression levels when the cells were treated with 10^−6^ M RA. When the treatment lasted 48 hours, there was no significant difference in the expression levels, between the two concentrations. The expression levels of Pdx1 were the same between the two time periods when the cells were treated with 10^−5^ M RA (Fig. 3A–C), however more dead cells appeared following 48 hours of treatment, as compared with 24 hours of treatment (data not shown).

**Figure 4:**
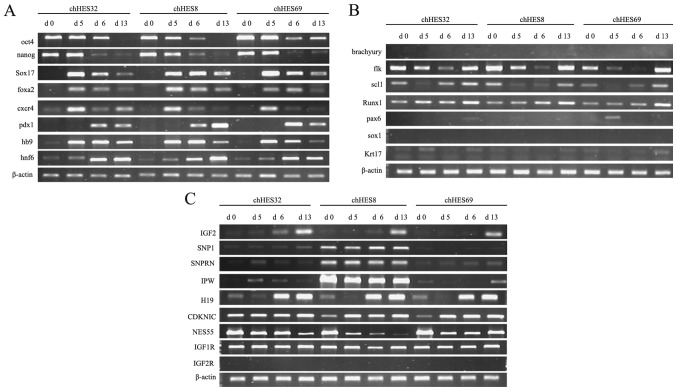
Expression of marker genes, mesoderm and ectoderm-related genes, and imprinted genes on days 0, 5, 6 and 13, as determined by quantitative polymerase chain reaction. (A) The expression levels of marker genes for embryonic stem cells (ESCs) (Oct4, Nanog), definitive endoderm (Sox17, Foxa2, Cxcr4) and pancreatic precursors (Pdx1, Hb9, Hnf6) in the three embryonic stem cell lines. (B) The expression levels of marker genes of the mesoderm (Brachyury, Flk, Scl1, Runx1) and ectoderm (Pax6, Sox1, Krt17) in the three embryonic stem cell lines. (C) The expression levels of paternally imprinted (IGF2, SNP1, SNPRN, IPW), maternally imprinted (H19, CDKNIC, NES55), and receptor (IGF1R, IGF2R) genes on days 5, 13 and 20, in the three embryonic stem cell lines. DE, defined endoderm; chHES8, normal human ESCs; chHES32, human parthenogenetic ESCs; chHES69, human parthenogenetic ESCs.

For further differentiation, 1% ITS and 5 μg/ml fibronectin was added to the medium for 7 days for the proliferation of pancreatic progenitors. It has previously been reported that Ex-4 enhanced the expression levels of Pdx1 and Ngn3 during β cell regeneration in STZ-treated mice (18), and 50 ng/ml Ex-4 was used for pancreatic hormone-expressing endocrine cell specification (19). Therefore, the present study selected Ex-4 as an accelerant for further differentiation of the pancreatic precursor cells. On day 13 Pdx1, the marker of pancreatic progenitors, was shown to be upregulated, and ~17.5±3.7% Pdx1 positive cells were detected (Fig. 3D and E). Furthermore, mesoderm-related genes including Flk1, were detected on day 13, however ectoderm-related genes, such as Krt17, were not detected (Fig. 4B). The imprinting gene, including IGF12 and H19 were active (Fig. 4C).

### Formation of ILCs

**Figure 5:**
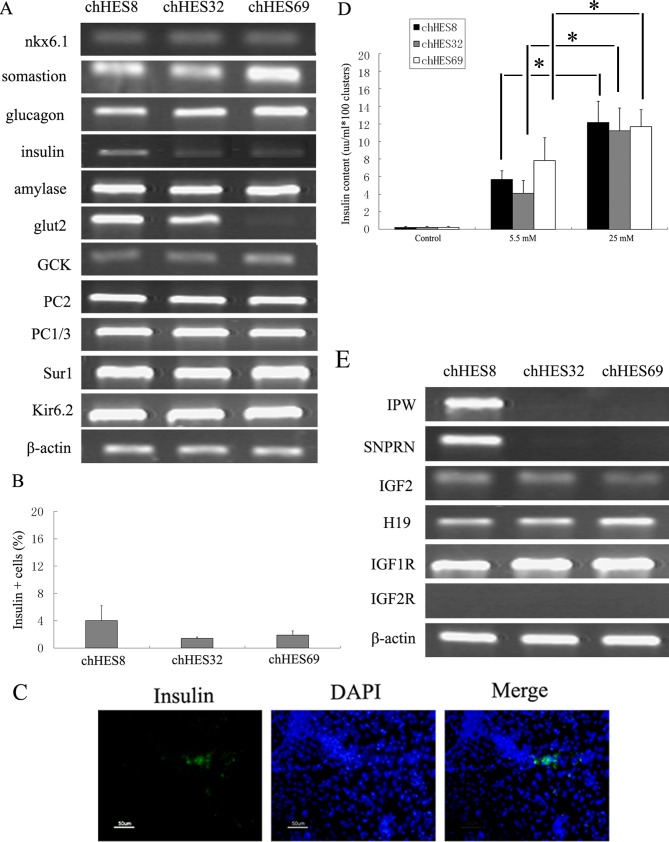
Identification of insulin producing cells. (A) Expression levels of imprinted genes and receptor genes on day 20 in the three embryonic stem cell lines, as determined by qPCR. (B) Expression levels of insulin on day 20. in the three embryonic stem cell lines. (C) Islets marker insulin was detected on day 20 by immunofluorescence analysis. Nuclei were stained with DAPI. Scale bar: 50 μm. (D) Insulan release in response to a glucose stimulus of 5.5 and 25mM in the three embryonic stem cell lines, as determined by an insulin release assay. (E) Expression levels of imprinted genes and receptor genes on day 20 in the theee embryonic stem cell lines, as determined by semi qPCR. Data are expressed as the mean ± standard deviation. Statistical analyses were performed using Student’s t-test. ^*^P<0.05. chHES8 normal human ESCs; chHES32 and chHES69, human parthenogenetic embryonic stem cells; qPCR, quantitative polymerase chain reaction.

On day 20, the pancreatic markers insulin, glucagon, somatostatin and the insulin secretion-related genes, Kir6.2 and PC1/3 were shown to be upregulated (Fig. 5A). The expression levels of these marker genes were consistent with the progression of pancreatic development. There were ~4.0±2.2% insulin-positive cells on day 20 (Fig. 5B and C). In the insulin release test, the culture medium prior to stimulation with glucose was used as the control sample. The insulin content in the control group was 0.02 μU/ml. Upon treatment of the cells with 5.5 and 25 mM glucose, the contents of insulin increased to 8.23±2.3 μU/ml and 17.36±2.4 μU/ml, respectively. The insulin content of the media was 1.5–4 times higher in the high glucose concentration group, as compared with the insulin content of the media obtained from the low glucose concentration group. The results indicated that the insulin release of the hpES-induced clusters corresponded to variations in the glucose concentration (Fig. 5D).

### Expression of imprinted genes and cell proliferation assay

Various imprinted genes were detected in the three ES cell lines. Paternally imprinted genes (SNP1, SNRPN, IPW) were detected in chHESC8, but not in chHESC32 and chHESC69 cells. Maternally imprinted genes (H19, CDKNIC, NES55) were expressed in all three of the ES cell lines. The IGF2 gene has been reported not to be expressed in hPESCs (8,20). However, in the present study, IGF2 was expressed in all three of the ES cell lines, with the expression being initiated on day 6 and lasting until day 20. The expression levels of IGF1R and IGF2R were also determined. Expression of IGF1R was observed in all of the ES cell lines, whereas IGF2R was not detected in any of the cell lines (Figs. 4C and 5E).

**Figure 6:**
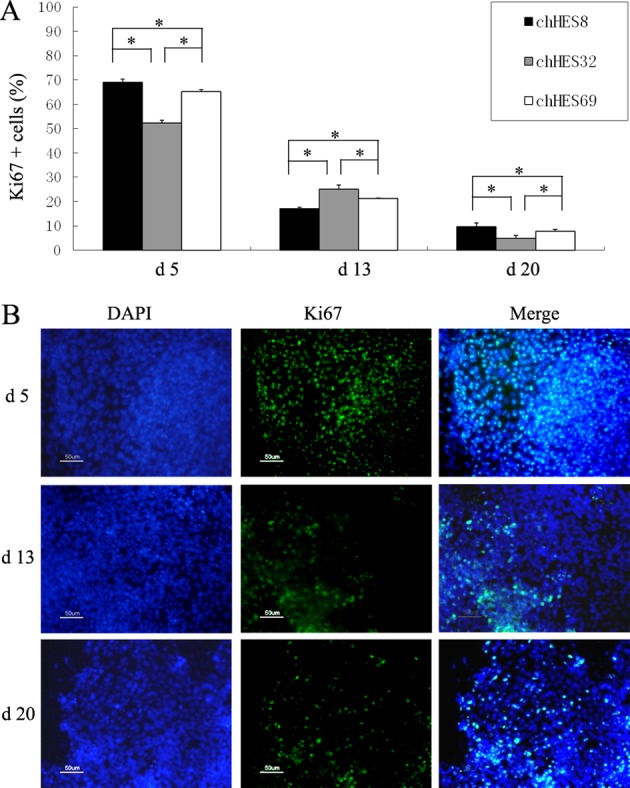
Proliferative ability assay. (A) Expression efficiency of Ki67 on days 5, 13 and 20, in the three embryonic stem cell lines. (B) Immunohistochemistry of the Ki67 protein on days 5, 13 and 20 visualized using DAPI staining techniques. chHES8, normal human ESCs; chHES32 and chHES69, human parthenogenetic embryonic stem cells. Scale bar: 50 μm. Data are expressed as the mean ± standard deviation. Statistical analyses were performed using Student’s t-test. *P<0.05.. chHES8 normal human ESCs; chHES32 and chHES69, human parthenogenetic embryonic stem cells.

Ki67 was chosen as the marker for the evaluation of the proliferative ability of hpESCs on days 5, 13, and 20. The results of the present study demonstrated that the proliferative ability of hpESCs reduced gradually, along with the extension of induction time. There were statistically significant differences between the three ES cell lines at the same stage of every differentiation (Fig. 6A and B).

## Discussion

Mammals can not reproduce by parthenogenesis due to the absence of paternal genetic material; the development of the mammalian embryo is dependent on the expression of the paternal genes. Embryological studies, using mouse parthenogenetic embryos, have demonstrated that they can only develop to an early stage, and that development arrests following 10 days gestation (21–24). In humans, a lack of some paternally imprinted genes will lead to developmental delay and mental retardation, manifesting in conditions including Prader-Willi Syndrome. Genetic imprinting has an important role in the progression of human development. However, there is currently no clear way of assessing the extent to which the expression and regulation of imprinted genes influences development, or the role of imprinted genes in differentiation, due to a lack of suitable *in vitro* research models. hpESCs offer a promising model for study *in vitro*. pESCs have a similar capacity for totipotency and proliferation as normal ESCs (25). The present study demonstrated that hpESCs can be induced into ILCs. These results support further study of pESCs and offer a feasible method for their introduction into a clinical setting.

For differentiation, Activin A was found to be a key factor in DE specification( 17), which acts in the same manner as the DE differentiation from hPESCs. A previous study reported that RA is important in the anteroposterior patterning of neuroectoderm and mesoderm in vertebrates (26). RA is also involved in the regulation of early pancreas bud formation and it is able to improve the expression of insulin in pancreatic β cells and in the INS-1 cell line (27,28). RA and FGF4 direct the differentiation of hESCs into foregut endoderm in a time- and concentration-dependent manner (29). In the present study, the initiation of pancreas progenitors was achieved by adding 10-6M RA for 48 h after a 10 h intermission following 5 days DE formation. The results demonstrated that the induced ILCs expressed islet-specific hormones and associated functional markers. Additionally, assessment of insulin release demonstrated that the degree of insulin release corresponded with alterations in glucose concentration. These results confirm that, following 20 days differentiation, functional insulin-producing cells were obtained from the hPESCs.

At a different differentiation stage, no statistically significant differences were observed in the expression level of the stage specific markers sox17, pdx1 and insulin among the three ES cell lines. However, the expression level of Ki67 was higher on day 5 and day 20 in the normal hESCs compared with the hPESCs. It was previously reported that the proliferative ability of partheno source fibroblasts was lower compared with the proliferative ability of normal fibroblasts (14). Whether hPESCs develop into functional cells and organs or not is associated with characteristics of gene imprinting (12). The paternally imprinted genes SNP1, SNRPN and IPW were detected in the chHESC8 cells, but not in the chHESC32 or chHESC69 cells. The maternally imprinted genes H19, CDKNIC and NES55 were expressed in all three of the ES cell lines. The expression of these imprinted genes was consistent with previously observed characteristics of hPESCs (8,20).

The present study found that the paternal gene IGF2 recovered its expression levels in hpESCs during the differentiation process, resulting in similar expression levels to normal ESCs. It has been observed that mouse β cells overexpressing IGF2 have higher levels of cell proliferation and islet hypertrophia (30). It has been suggested that IGF2 may be important in the determination of endocrine cell fate, proliferative ability and amylon metabolism in the perinatal period (31). A previous study compared the gene expression levels of IGF2, H19, IGF2R and SNRPN in androgenetic ESCs, pESCs and normal ESCs (20). The expression levels of these genes in pESCs and androgenetic ESCs was found to differ from the normal ESCs. The markers of genomic imprinting were lost in the somatic cells of the parthenogenetic embryonic chimera, but were maintained in its stem cells. The loss of imprinting markers correlates with the expression levels of imprinted genes (32). Other studies have also suggested that the biallele methylation difference of IGF2 is established gradually throughout development, including the postnatal period. Previous data have revealed that the expression of IGF2 is monallelic in the fetal stage of human and mouse development (33, 34). In the human IGF2 gene, there are four independent promoters switching on allele expression in the paternal chromosome in an individual developmental period (33–35). Following birth, the distal promoter of the human hepatic IGF2 was shown to be active on both chromosomes, which leads to biallelic expression in adults. It was presumed, in the present study, that promoters of maternal IGF2 were activated during the differentiation of hpESCs. IGF2 promoter activity may be influenced by the external environment, resulting in the recovery of the absent IGF2 expression and the ensuing effect of IGF2 in pancreatic development.

In conclusion, parthenogenetic activation of human oocytes is a novel method and may be used to produce histocompatible cells for cell-based therapy. Furthermore, stem cells derived from human parthenogenetic embryos could alleviate some of the ethical concerns over embryonic and stem cell research, and hpESCs provide a valuable *in vitro* model to explore the influence of imprinted genes on cell differentiation. It will be important to confirm the safety of this treatment at the epigenetic level, prior to formal clinical application (36).

## Figures and Tables

**Table I:** Primer sequences used in the quantitative polymerase chain reaction

Gene	Size (bp)	Sequence of primer (5′-3′)	Temp (°C)	Cycles
Sox17	292	F: GGCGCAGCAGAATCCAGA		
		R: CCACGACTTGCCCAGCAT	58.0	30
Foxa2	588	F: GGGAGCGGTGAAGATGGA		
		R: TCATGTTGCTCACGGAGGAGTA	57.5	30
Pdx1	217	F: GGATGAAGTCTACCAAAGCTCACGC		
		R: CCAGATCTTGATGTGTCTCTCGGTC	65.0	30
Insulin	244	F: CAGTGACCTGTCTTGGTTTTCCG		
		R: CAGCCGAGTAGTTTTCATCATTGC	65.0	30
Glucagon	307	F: AGGCAGACCCACTCAGTGA		
		R: AACAATGGCGACCTCTTCTG	55.0	30
Somatostatin	179	F: GTACTTCTTGGCAGAGCTGCTG		
		R: CAGAAGAAATTCTTGCAGCCAG	55.0	30
Amylase	358	F: CTGACAACTTCAAAGCAAA		
		R: TACAGCATCCACATAAATACGA	57.0	30
GCK	376	F: AGGGAATGCTTGCCGACTC		
		R: CACTGGCCTCTTCATGGGT	57.1	30
PC2	314	F: GCATCAAGCACAGACCTACACTCG		
		R: GAGACACAACCACCCTTCATCCTTC	60.5	30
PC1/3	456	F: TTGGCTGAAAGAGAACGGGATACATCT		
		R: ACTTCTTTGGTGATTGCTTTGGCGGTG	65.4	30
Kir6.2	499	F: CGCTGGTGGACCTCAAGTGGC		
		R: CCTCGGGCTGGTGGTCTTGCG	65.0	30
SUR1	429	F: GTGCACATCCACCACAGCACATGGCTTC		
		R: GTGTCTTGAAGAAGATGTATCTCCTCAC	62.1	30
Oct3/4	168	F: CTTGCTGCAGAAGTGGGTGGAGGAA		
		R: CTGCAGTGTGGGTTTCGGGCA	64.0	28
Nanog	387	F: ACTGTCTCTCCTCTTCCCTCCTCC		
		R: GTAGAGGCTGGGGTAGGTAGGTG	64.0	28
Hb9	403	F: GCGCTCTCCTACTCGTACCC		
		R: CTTCTGTTTCTCCGCTTCCTG	60.9	35
Hnf6	457	F: AGTAATTCAGGGCAGATGGAAG		
		R: CGTTCATGAAGAAGTTGCTGAC	56.0	35
Cxcr4	80	F: CACCGCATCTGGAGAACCA		
		R: GCCCATTTCCTCGGTGTAGTT	50.0	28
Sox1	464	F: CAATGCGGGGAGGAGAAGTC		
		R: CTCTGGACCAAACTGTGGCG	53.0	30
Krt17	119	F: GGAGATTGCCACCTACCG		
		R: TGCCATCCTGGACCTCTT	60.0	30
Brachyury	329	F: ACCCAGTTCATAGCGGTAGC		
		R: CAATTGTCATGGGATTCAG	55.0	30
FLK	721	F: GAGGGCCACTCATGGTGATTGT		
		R: TGCCAGCAGTCCAGCATGGTCTG	55.0	28
Scl1	331	F: ATGGTGCAGCTGACTCCTCC		
		R: TCTCATTCTTGCTGAGCTTC	55.0	35
Runx1	516	F: CAGTGACCTGTCTTGGTTTTCCG		
		R: CAGCCGAGTAGTTTTCATCATTGC	60.0	35
IGF2	86	F: TCCCCTGATTGCTCTACCCA		
		R: GCAGTTTTGCTCACTTCCGATT	58.0	28
KCNQ10STSNP1	466	F: CAGCCACCTCTGTGGCGTGAATGTTCT		
		R: GCTCAAACCCGTCTCTGAAATGCACGG	55.0	35
SNRPN	112	F: TGGCACCTTTAAGGCTTTTG		
		R: CCGCTTTTCTTCACGCTCT	58.0	28
IPW	868	F: GGGAACTCTTCTGGGAGTGAATGTTATCA		
		R: GGGAGGTTCATTGCACAGAAATTTGG	55.0	28
H19	142	F: CCGGACACAAAACCCTCTAGCT		
		R: TGTTCCGATGGTGTCTTTGATG	58.0	28
CDKNIC	146	F: TGGGACCGTTCATGTAGC		
		R: GGACCAGTGTACCTTCTCG	50.5	28
NES55	1141	F: TCGGAATCTGACCACGAGCA		
		R: CACGAAGATGATGGCAGTCAC	55.0	35
IGF1R	540	F: GAATGGAGTGCTGTATGCCTCTGTGAACC		
		R: GTGAAATCTTCGGCTACCATGCAATTCCG	55.0	28
IGF2R	284	F: GTTGTCTGCCCTCCAAAGAA		
		R: CCTTTGGAGTACGTGACAAG	55.0	28
β-actin	200	F: CGCACCACTGGCATTGTCAT		
		R: TTCTCCTTGATGTCACGCAC	60.0	28

Bp, base pairs; temp, temperature; F, forward; R, reverse.
